# SARM1 Ablation Is Protective and Preserves Spatial Vision in an In Vivo Mouse Model of Retinal Ganglion Cell Degeneration

**DOI:** 10.3390/ijms23031606

**Published:** 2022-01-30

**Authors:** Laura K. Finnegan, Naomi Chadderton, Paul F. Kenna, Arpad Palfi, Michael Carty, Andrew G. Bowie, Sophia Millington-Ward, G. Jane Farrar

**Affiliations:** 1 Department of Genetics, The School of Genetics and Microbiology, Trinity College Dublin, D02 VF25 Dublin, Ireland; chaddern@tcd.ie (N.C.); paul.kenna@tcd.ie (P.F.K.); palfia@tcd.ie (A.P.); sophia@maths.tcd.ie (S.M.-W.); jane.farrar@tcd.ie (G.J.F.); 2 The Research Foundation, Royal Victoria Eye and Ear Hospital, D02 XK51 Dublin, Ireland; 3 Trinity Biomedical Sciences Institute, The School of Biochemistry and Immunology, Trinity College Dublin, D02 R590 Dublin, Ireland; cartymi@tcd.ie (M.C.); agbowie@tcd.ie (A.G.B.)

**Keywords:** axon degeneration, sterile alpha and Toll/interleukin-1 receptor motif-containing 1 (SARM1), NAD+, NADase, retinal degeneration

## Abstract

The challenge of developing gene therapies for genetic forms of blindness is heightened by the heterogeneity of these conditions. However, mechanistic commonalities indicate key pathways that may be targeted in a gene-independent approach. Mitochondrial dysfunction and axon degeneration are common features of many neurodegenerative conditions including retinal degenerations. Here we explore the neuroprotective effect afforded by the absence of sterile alpha and Toll/interleukin-1 receptor motif-containing 1 (SARM1), a prodegenerative NADase, in a rotenone-induced mouse model of retinal ganglion cell loss and visual dysfunction. *Sarm1* knockout mice retain visual function after rotenone insult, displaying preservation of photopic negative response following rotenone treatment in addition to significantly higher optokinetic response measurements than wild type mice following rotenone. Protection of spatial vision is sustained over time in both sexes and is accompanied by increased RGC survival and additionally preservation of axonal density in optic nerves of *Sarm1^−/−^* mice insulted with rotenone. Primary fibroblasts extracted from *Sarm1^−/−^* mice demonstrate an increased oxygen consumption rate relative to those from wild type mice, with significantly higher basal, maximal and spare respiratory capacity. Collectively, our data indicate that *Sarm1* ablation increases mitochondrial bioenergetics and confers histological and functional protection in vivo in the mouse retina against mitochondrial dysfunction, a hallmark of many neurodegenerative conditions including a variety of ocular disorders.

## 1. Introduction

Retinal ganglion cells (RGCs) constitute approximately 1% of retinal cells but are vital for vision, integrating visual information from the retinal layers and transmitting this information to the brain via the optic nerve. When RGCs or their axons, which form the optic nerve, are damaged and degenerate, vision is compromised or lost. These cells are affected in many degenerative conditions, including glaucoma, Leber hereditary optic neuropathy (LHON) and Parkinson’s and Alzheimer’s diseases [1,2,3]. Although these diseases have different mechanisms of action—glaucoma can involve increased pressure on RGCs leading to axonal degeneration and cell death, while LHON is a mitochondrially inherited neuropathy that primarily affects RGCs—protection against the death of these cells could, in principle, be beneficial in many disease settings. Here we demonstrate protection against RGC death and vision loss through ablation of sterile alpha and Toll/interleukin-1 receptor motif-containing 1 (SARM1), a prodegenerative NADase.

Optic neuropathy is an umbrella term for diseases involving optic nerve damage and subsequent axon degeneration and neuronal death. Attractive therapies for optic neuropathies would ideally protect the soma and axon from degeneration. Indeed, soma death and axon degeneration are distinct from one another [4,5,6]. Notably, some optic neuropathies, such as optic neuritis, can benefit from treatment to reverse vision loss before RGC death has occurred [7,8]. In such situations, protecting the injured optic nerve from degeneration, not only can the connection from the retina to the brain be maintained, but progression to RGC death may also be prevented or delayed.

In recent years, the mechanisms underlying Wallerian degeneration, an injury-induced process of anterograde axon degeneration, have been elucidated, yielding promising targets for axon protection. Nerve transection and nerve crush models have traditionally been used to study Wallerian degeneration, but chemical insults have also been shown to provoke a degeneration akin to Wallerian degeneration [9,10,11]. Axon degeneration in neurodegenerative diseases follows the same pathway, however, instead of being severed, the axons progressively degenerate in a “dying back” process. Axon degeneration is distinct from apoptosis, with inhibitors of apoptosis failing to block Wallerian degeneration, and an absence of caspase activation in axon degeneration [4,5,12,13,14,15].

Wallerian degeneration involves an active programme of disassembly of the injured axon [16,17,18]. Genetic screens identified SARM1 as a prodegenerative factor whose ablation slowed the progression of Wallerian degeneration in dorsal root ganglia and sciatic nerves [15,19]. SARM1 is a highly conserved member of the TIR adapter family, with roles in innate immunity and response to infection [20,21,22,23]. The TIR domain of SARM1 has intrinsic NADase activity, cleaving NAD+ to produce nicotinamide, adenosine diphosphate ribose (ADPR) and cyclic ADPR (cADPR) [24]. This domain is required to promote degeneration [15,25,26]. NAD+ has been shown to be protective against axon degeneration in vitro and in vivo [15,27,28]. Forced dimerisation of the TIR domains alone is sufficient to induce rapid NAD+ loss, and NAD+ depletion in *Sarm1*^−/−^ dorsal root ganglion cells is sufficient to induce axon degeneration, reinforcing the role of the TIR domain as executioner [29]. The ARM domain interacts with the TIR domain to lock SARM1 in an inactive conformation. This lock site has binding sites for NAD+ and NMN, with NAD+ stabilising the lock. As NAD+ decreases, the ARM-TIR lock breaks and TIR domains can interact, activating SARM1 to deplete NAD+ further [30,31,32].

Suppression of SARM1 has been explored as a mechanism to protect against axon degeneration in several disease settings, including models of photoreceptor degeneration, optic nerve crush, peripheral neuropathy and diabetic neuropathy [33,34,35,36,37]. Notably, GWAS have flagged *SARM1* variants in connection with amyotrophic lateral sclerosis (ALS), and increased activity of SARM1 has been reported in iPSC-derived neurons from Parkinson’s patients [38,39,40].

Mitochondrial depolarisation and disruption of the electron transport chain have been shown to induce axon degeneration and cell death in neurons, but in vitro *Sarm1*^−/−^ superior cervical ganglion cells and dorsal root ganglion cells are strongly resistant to this [14,41,42]. Since many retinal degenerations feature mitochondrial dysfunction due to pathogenic variants directly affecting mitochondrial function, or a build-up of reactive oxygen species (ROS), mitigating mitochondrial stress is attractive from a therapeutic standpoint. Rotenone has been used to model mitochondrial dysfunction in several neurodegenerative diseases, from Parkinson’s disease to LHON [43,44,45,46]. Rotenone inhibits complex I of the electron transport chain, preventing mitochondrial respiration and leading to generation of reactive oxygen species [47,48,49]. SARM1 can be activated by oxidative stressors such as rotenone [40,50,51], with in vitro studies suggesting that *Sarm1*^−/−^ dorsal root ganglion cells may be protected from rotenone-induced degeneration [14,42].

In this study, to interrogate the potential therapeutic value of SARM1 depletion in vivo, a rotenone-induced mouse model of RGC loss and visual dysfunction has been employed. Critically, genetic ablation of *Sarm1* is shown to protect against rotenone-induced loss of visual function in vivo. While wild type mice receiving rotenone intravitreally suffer significant loss of photopic negative response and spatial vision, *Sarm1*^−^^/−^ mice do not. The benefit provided by *Sarm1* ablation is observed in both sexes. In addition, while both wild type and *Sarm1*^−/−^ mice present with loss of RGCs in response to rotenone insult, there is increased RGC density in retinas of mice lacking SARM1. These mice also display significant protection of RGC axons of the optic nerve, relative to wild type mice, indicating that *Sarm1* ablation is protective against optic nerve degeneration. Despite some RGC loss, the functional protection afforded by *Sarm1* ablation is preserved over time, underscoring the importance of the maintenance of connectivity for preservation of function. In addition, fibroblasts extracted from *Sarm1*^−/−^ mice display improved mitochondrial function as measured by oxygen consumption rate (OCR), directly reflective of the function of the electron transport chain. Primary *Sarm1*^−/−^ fibroblasts demonstrate increased OCR relative to wild type fibroblasts, with significantly higher basal OCR, spare respiratory capacity (SRC) and maximal respiration as well as ATP production. Our data indicate that deletion of *Sarm1* confers functional protection against rotenone insult and increases mitochondrial bioenergetics.

## 2. Results

The objective of the study was to evaluate whether ablation of SARM1, a key component of the axon degeneration pathway, could protect against complex I deficiency, mitochondrial dysfunction and subsequent vision loss in a well-established chemically induced mouse model of retinal degeneration [44,45,46]. Intravitreal injection of rotenone, a complex I inhibitor, enabled disruption of mitochondrial function and induction of oxidative stress in RGCs, whose axons relay signals integrated from the retinal layers to the visual cortex. We hypothesised that deficiency in the axon destructive *Sarm1* gene may protect RGCs from rotenone-induced degeneration, maintaining connectivity between the retina and the brain and preserving vision.

### 2.1. Protection against Loss of Spatial Vision

**Figure 1:**
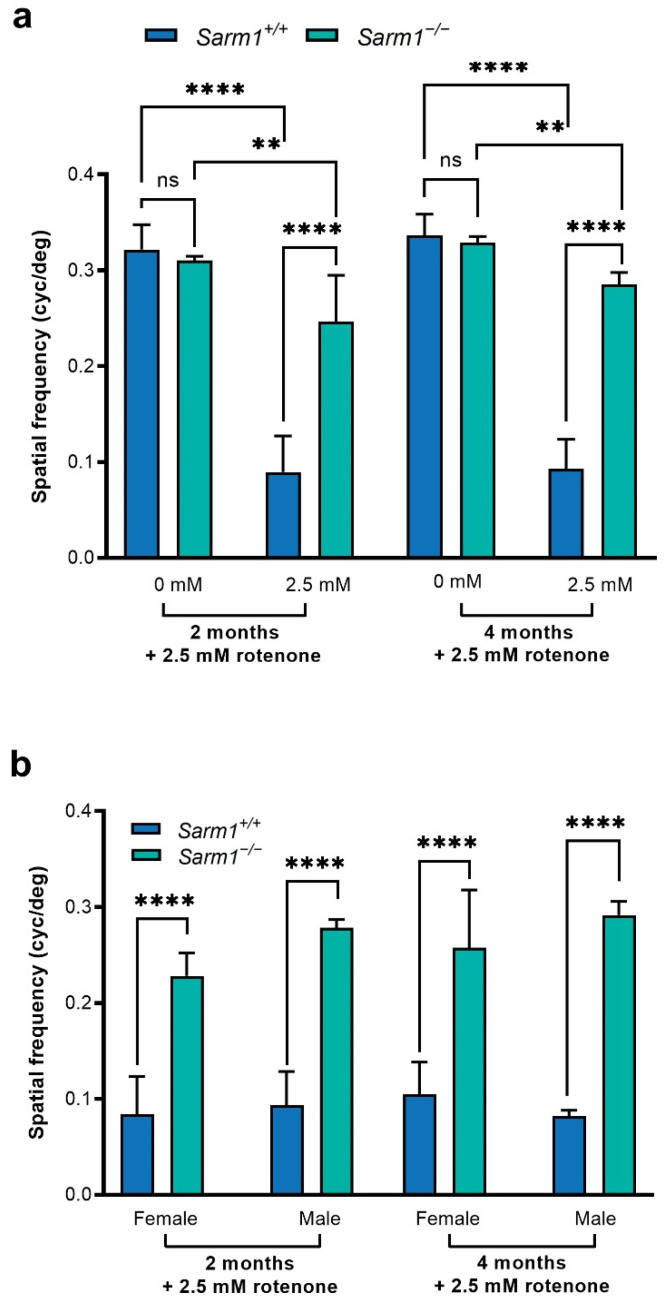
Spatial vision following rotenone insult. Rotenone was delivered bilaterally via intravitreal injection. Optokinetic responses were measured 2 months and 4 months post-injection using an OptoMotry virtual optokinetic system (Cerebral Mechanics). Bar charts represent the mean combined spatial frequency threshold. Error bars represent SD, ** *p* < 0.01, **** *p* < 0.0001. (**a**) *Sarm1*^−/−^ mice retained a higher spatial frequency threshold following rotenone treatment compared to wild type mice. This was sustained over time (0.25 ± 0.05 cyc/deg vs. 0.09 ± 0.04 cyc/deg at 2 months post-injection; 0.29 ± 0.01 cyc/deg vs. 0.09 ± 0.03 cyc/deg at 4 months post-injection). (**b**) Spatial vision was preserved in both sexes over time (2 and 4 months post-injection), with no significant differences between the sexes within genotypes (2 months post-rotenone: female *Sarm1^+/+^* 0.08 ± 0.04 cyc/deg vs. female *Sarm1*^−/−^ 0.23 ± 0.02 cyc/deg, *p* < 0.0001; male *Sarm1^+/+^* 0.11 ± 0.03 cyc/deg vs. male *Sarm1*^−/−^ 0.26 ± 0.06, *p <* 0.0001. 4 months post-rotenone: female *Sarm1^+/+^* 0.09 ± 0.03 cyc/deg vs. female *Sarm1*^−/−^ 0.28 ± 0.01 cyc/deg; *p <* 0.0001, male *Sarm1^+/+^* 0.08 ± 0.01 cyc/deg vs. male *Sarm1*^−/−^ 0.29 ± 0.01 cyc/deg, *p* < 0.0001).

We evaluated whether ablation of SARM1 protected against the decline in spatial vision that occurs following loss of RGCs. Age-matched adult C57BL/6J (*n* = 11) and *Sarm1*^−/−^ (*n* = 11) mice received 3 µL rotenone (2.5 mM) in each eye, administered intravitreally. Control C57BL/6J *Sarm1^+/+^* (*n =* 4) and additional *Sarm1*^−/−^ (*n =* 7) mice received no rotenone. Two months and four months post-injection, OKRs were measured. The spatial frequency threshold—the point at which the mouse ceases to track the moving sine wave grating—was measured for each eye individually before being combined to obtain a combined spatial frequency threshold for each animal. At each time point, 3–4 readings were taken for each animal. Wild type *Sarm1^+/+^* and *Sarm1*^−/−^ mice that did not receive rotenone had indistinguishable OKR responses (2 month time point: 0.32 ± 0.03 cyc/deg vs. 0.31 ± 0.04 cyc/deg; 4 month time point: 0.34 ± 0.02 cyc/deg vs. 0.33 ± 0.01 cyc/deg; Figure 1a). Notably, *Sarm1*^−/−^ mice receiving rotenone displayed significantly higher OKRs than their wild type counterparts receiving rotenone (2 months post-rotenone: 0.25 ± 0.05 cyc/deg vs. 0.09 ± 0.04 cyc/deg, *p* < 0.0001; 4 months post-rotenone: 0.29 ± 0.012 cyc/deg vs. 0.09 ± 0.03 cyc/deg; *p* < 0.0001). Indeed, the OKRs of *Sarm1*^−/−^ mice receiving rotenone were more similar to those of uninjected *Sarm1*^−/−^ and wild type *Sarm1^+/+^* mice without rotenone than they were to OKRs of wild type mice receiving rotenone (2 months post-rotenone: rotenone-insulted *Sarm1*^−/−^ 0.25 ± 0.05 cyc/deg vs. uninjected *Sarm1*^−/−^ 0.31 ± 0.04 cyc/deg, uninjected *Sarm1*^+/+^ 0.32 ± 0.03 cyc/deg, rotenone-insulted *Sarm1*^+/+^ 0.09 ± 0.04 cyc/deg. 4 months post-rotenone: rotenone-insulted *Sarm1*^−/−^ 0.29 ± 0.01 cyc/deg vs. uninjected *Sarm1*^−/−^ 0.33 ± 0.01 cyc/deg, uninjected *Sarm1*^+/+^ 0.34 ± 0.02 cyc/deg, rotenone-insulted *Sarm1*^+/+^ 0.09 ± 0.03 cyc/deg). Furthermore, as several retinal degenerations and indeed many mitochondrial dysfunction disorders have a sex bias associated with their clinical manifestation [52,53,54,55,56], we compared males (*n =* 3 wild type; *n =* 6 *Sarm1*^−/−^) and females (*n =* 8 wild type; *n =* 5 *Sarm1*^−/−^) separately to assess the possibility of a sex bias in our model. The absence of SARM1 provided benefit and protected against rotenone-induced decay of spatial frequency threshold in both sexes, with no significant differences between the sexes within genotypes observed (2 months post-rotenone: female *Sarm1^+/+^* 0.08 ± 0.04 cyc/deg vs. female *Sarm1*^−/−^ 0.23 ± 0.02 cyc/deg, *p* < 0.0001; male *Sarm1^+/+^* 0.11 ± 0.03 cyc/deg vs. male *Sarm1*^−/−^ 0.26 ± 0.06, *p* < 0.0001. 4 months post-rotenone: female *Sarm1^+/+^* 0.09 ± 0.03 cyc/deg vs. female *Sarm1*^−/−^ 0.28 ± 0.01cyc/deg, *p* < 0.0001; male *Sarm1^+/+^* 0.08 ± 0.01 cyc/deg vs. male *Sarm1*^−/−^ 0.29 ± 0.01 cyc/deg, *p* < 0.0001; Figure 1b).

**Figure 2:**
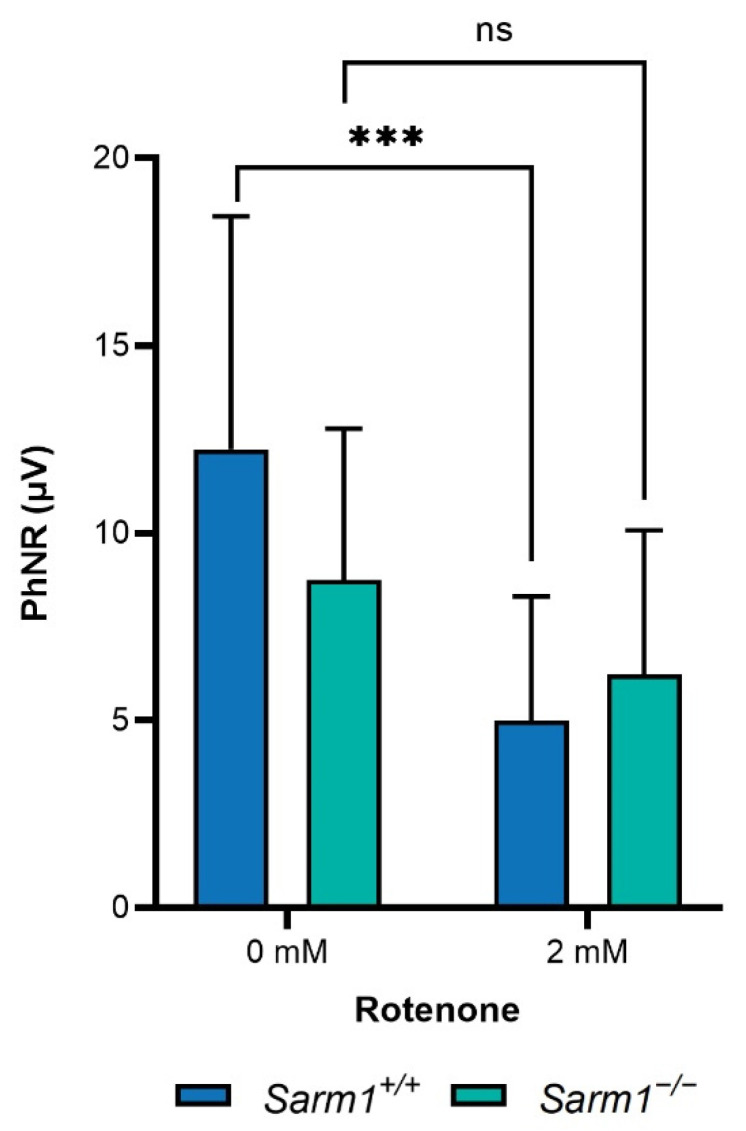
Analysis of PhNR. Rotenone was delivered via intravitreal injection. PhNRs were measured six weeks post-injection. Bar charts represent the mean PhNR. Error bars represent SD, *** *p* < 0.001, ns = non-significant. Reduction in PhNR following rotenone treatment was significantly greater in wild type *Sarm1*^+/+^ mice (12.23 ± 6.22 μV vs. 5.01 ± 3.3 μV; *p* < 0.001) than *Sarm1*^−/−^ mice, where the reduction was not significant (8.75 ± 4.04 μV vs. 6.23 ± 3.85 μV).

The photopic negative response (PhNR) provides an electrophysiological measure of RGC activity. Adult C57BL/6J (*n* = 11) and *Sarm1*^−/−^ (*n* = 13) mice received 3 µL rotenone (2 mM) in one eye only, administered intravitreally. Six weeks following rotenone treatment, PhNRs were measured. While there was a decrease in PhNR in both genotypes following rotenone treatment, this was only significant in wild type mice (12.23 ± 6.22 μV vs. 5.01 ± 3.3 μV; *p* < 0.001). There was a smaller, non-significant decrease in PhNR in *Sarm1*^−/−^ mice following rotenone treatment (8.75 ± 4.04 μV vs. 6.23 ± 3.85 μV; Figure 2).

### 2.2. Protection of RGCs and Their Axons

**Figure 3:**
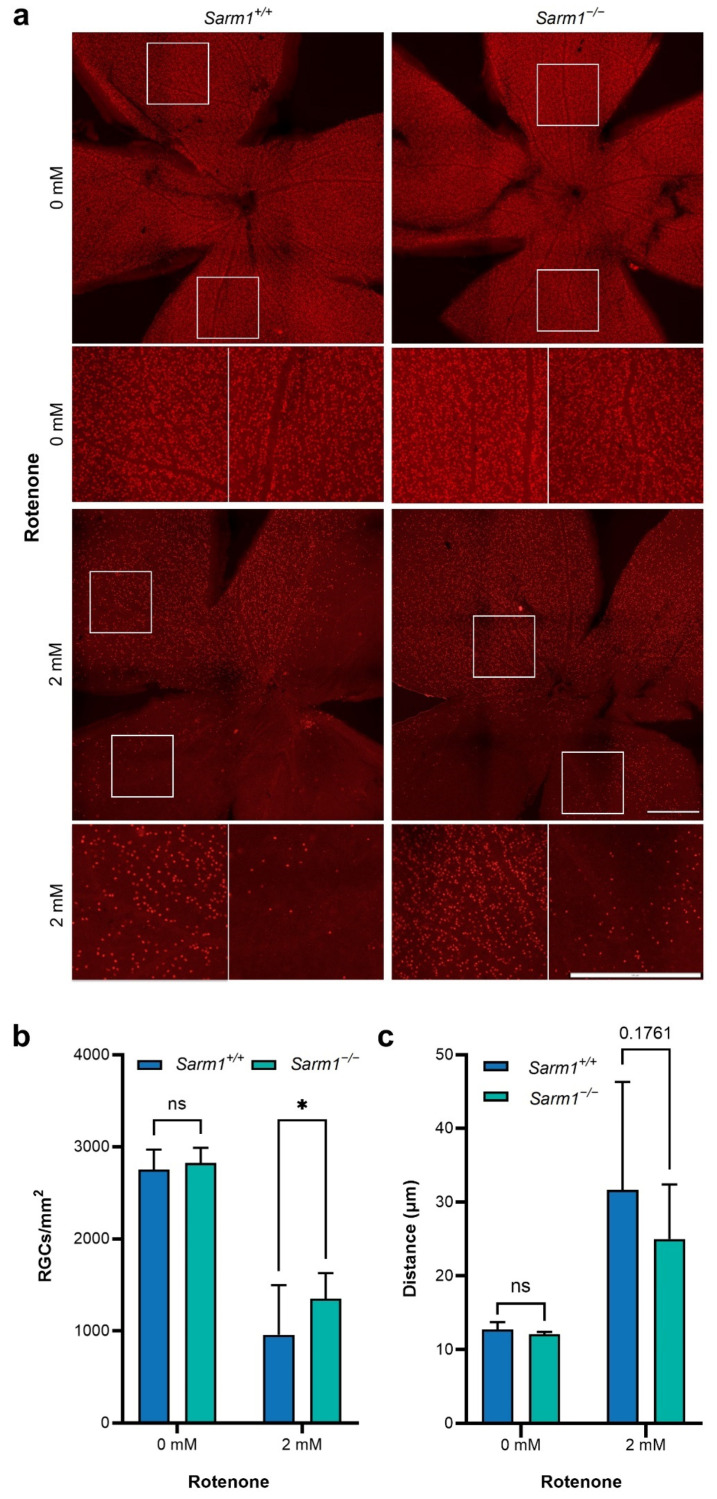
Quantification of retinal ganglion cells. Two months after intravitreal injection of rotenone, eyes were enucleated, fixed and dissected. Retinas were stained for BRN3A and wholemounted. Representative images of each retina are denoted by white squares. Scale bars = 500 µm (**a**). (**b**) Labelled RGCs were quantified using Olympus CellSens software. Number of RGCs is significantly reduced in both genotypes following rotenone treatment (*Sarm1^+/+^* 2752 ± 215 RGCs/mm^2^ vs. 959 ± 537 RGCs/mm^2^; *Sarm1*^−/−^ 2824 ± 164 RGCs/mm^2^ vs. 1352 ± 275 RGCs/mm^2^ *p* < 0.0001). There were significantly more RGCs in *Sarm1*^−/−^ retinas following rotenone treatment (*n =* 11) relative to wild type (*n =* 10; 1352 ± 275 RGCs/mm^2^ vs. 959 ± 537 RGCs/mm^2^; *p* < 0.05). (**c**) Mean distance between RGCs in rotenone-treated retinas was calculated using the BioVoxxel toolbox on ImageJ. Each bar represents the average mean distance between neighbouring RGCs, (*n =* 9–11). There was a trend towards decreased distance between *Sarm1*^−/−^ RGCs relative to wild type (24.99 ± 7.39 μm, range 20.51 μm vs. 31.65 ± 14.66 μm, range 41.87 μm; *p* = 0.1761). Error bars (**b**,**c**) represent SD values and * *p* < 0.05, ns = non-significant.

Adult mice were injected intravitreally with 2 mM rotenone. Two months post rotenone administration, animals were sacrificed and eyes enucleated. To examine the effect of rotenone insult on RGC death, retinas from each genotype were stained for BRN3A, an RGC-specific cell marker, and whole-mounted (Figure 3a). RGC counts were performed on uninjected eyes (*n =* 10 for wild type; *n =* 12 for *Sarm1*^−/−^) and rotenone-treated eyes (*n =* 10 for wild type; *n =* 11 for *Sarm1*^−/−^) at two months post-injection. There was significant RGC loss following rotenone treatment in both genotypes (*Sarm1^+/+^* 2752 ± 215 RGCs/mm^2^ vs. 959 ± 537 RGCs/mm^2^; *Sarm1*^−/−^ 2824 ± 164 RGC s/mm^2^ vs. 1352 ± 275 RGCs/mm^2^*, p* < 0.0001; Figure 3b). However, *Sarm1*^−/−^ retinas treated with rotenone had significantly higher RGC density than wild type mouse retinas (1352 ± 275 RGCs/mm^2^ vs. 959 ± 537 RGCs/mm^2^; *p* < 0.05). While mice of both genotypes suffered significant loss of RGCs after intravitreal rotenone treatment, *Sarm1*^−/−^ retinas both retained greater RGCs density and displayed a trend towards a more even distribution of remaining RGCs across the retina compared to wild type counterparts. To quantify the distribution of RGCs across the retina, distances between RGCs were calculated using the 2D Particle Distribution tool of the BioVoxxel toolbox for ImageJ [57]. 4–8 areas per retina were analysed, accounting for 70–80% of the retina. Distance between cells was not expected to vary significantly if cells were evenly distributed, whereas if within a retina the distribution of RGCs was uneven, the distances between cells would vary more substantially within the retina (Figure 3c). We found a trend towards decreased distance between RGCs in *Sarm1*^−/−^ retinas treated with rotenone compared with wild type retinas treated with rotenone (24.99 ± 7.39 μm, range 20.51 μm vs. 31.65 ± 14.66 μm, range 41.87 μm; *p* = 0.18).

**Figure 4:**
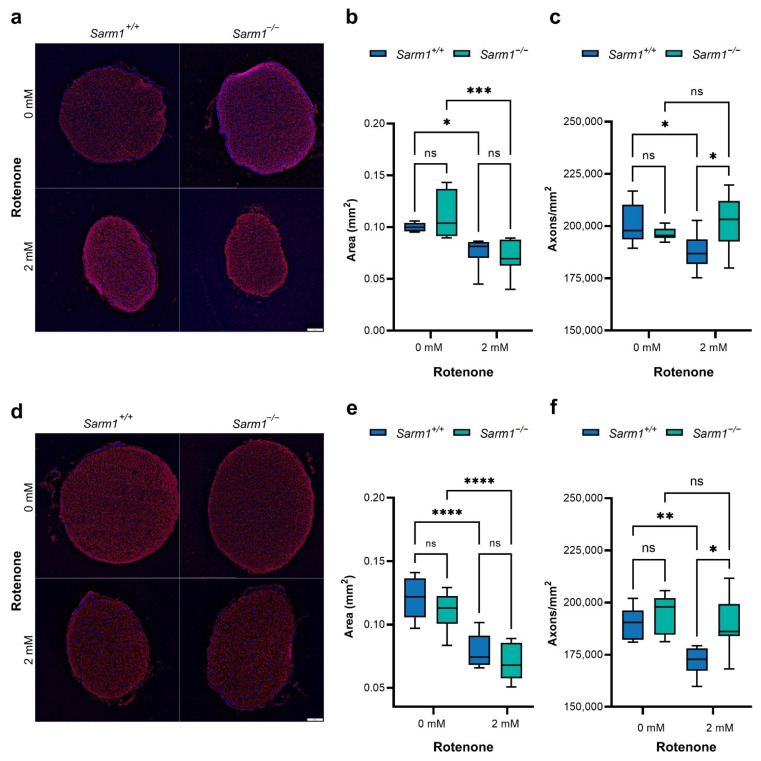
Quantification of axons. Two months after intravitreal injection of rotenone, eyes were enucleated, fixed and dissected. Optic nerves were cryosectioned (7 μm) and stained for NF200 (red). Nuclei were stained with DAPI (blue). Segments of the proximal (**a**) and distal (**d**) optic nerve, the portion of the nerve nearest the eye and brain respectively, were sectioned and analysed separately. Scale bars (**a**,**d**) = 50 µm. (**b**,**c**,**e**,**f**) Axons and optic nerve area were quantified using Olympus CellSens software. Optic nerves were thinner following rotenone insult in both genotypes (*n* = 6–10), both close to the eye ((**b**); *Sarm1^+/+^* 0.10 ± 0.004 mm^2^ vs. 0.07 ± 0.02 mm^2^, *p* < 0.05; *Sarm1*^−/−^ 0.11 ± 0.02 mm^2^ vs. 0.07 ± 0.02 mm^2^, *p* < 0.001) and brain ((**e**) *Sarm1^+/+^* 0.12 ± 0.02 mm^2^ vs. 0.08 ± 0.01 mm^2^, *p* < 0.0001, *Sarm1*^−/−^ 0.11 ± 0.02 mm^2^ vs. 0.07 ± 0.01 mm^2^, *p* < 0.0001). Density of axons was protected in *Sarm1*^−/−^ optic nerves, with no significant difference between insulted and non-insulted sections from proximal ((**c**) 201,631 ± 12,619 axons/mm^2^ vs. 196,265 ± 3030 axons/mm^2^) or distal ((**f**) 188,764 ± 13,580 axons/mm^2^ vs. 194,208 ± 9417 axons/mm^2^) *Sarm1*^−/−^ optic nerves. There was a significant reduction in axonal density in wild type optic nerves receiving rotenone relative to both non-insulted *Sarm1^+/+^* and insulted *Sarm1*^−/−^ optic nerves (*n* = 6–10; Proximal: rotenone-insulted *Sarm1^+/+^* 187,926 ± 7851 axons/mm^2^ vs. uninjected *Sarm1*^+/+^ 200,835 ± 9874 axons/mm^2^ *p <* 0.05; rotenone-insulted *Sarm1^+/+^* 187,926 ± 7851 axons/mm^2^ vs. rotenone-insulted *Sarm1*^−/−^ 201,631 ± 12,619 axons/mm^2^, *p <* 0.05. Distal: rotenone-insulted *Sarm1^+/+^* 172,002 ± 7086 axons/mm^2^ vs. uninjected *Sarm1*^+/+^ 190,789 ± 7402 axons/mm^2^, *p <* 0.01; rotenone-insulted *Sarm1^+/+^* 172,002 ± 7086 axons/mm^2^ vs. rotenone-insulted *Sarm1*^−/−^ 188,764 ± 13,580 axons/mm^2^, *p <* 0.05). Error bars (**b**–**c**,**e**–**f**) represent SD values, * *p* < 0.05, ** *p* < 0.01 and *** *p* < 0.001, **** *p* < 0.0001, ns = non-significant.

Given the role of SARM1 in axon degeneration, optic nerves were analysed to assess whether ablation of SARM1 protects against degeneration of RGC axons. Two months after rotenone administration, optic nerves were cryosectioned and stained for Neurofilament 200 (NF200). Sections from the proximal and distal optic nerve, defined as the portion of the nerve close to the eye and brain respectively, were analysed separately. Quantification was performed on 3–5 sections per sample, with *n* = 6–10 optic nerves per group. Both proximal (Figure 4b) and distal (Figure 4e) optic nerve thickness was reduced in both genotypes following rotenone treatment (Proximal: *Sarm1^+/+^* 0.10 ± 0.004 mm^2^ vs. 0.08 ± 0.02 mm^2^, *p* < 0.05, *Sarm1*^−/−^ 0.11 ± 0.02 mm^2^ vs. 0.07 ± 0.02 mm^2^, *p* < 0.001; Distal: *Sarm1^+/+^* 0.12 ± 0.02 mm^2^ vs. 0.08 ± 0.01 mm^2^, *p* < 0.0001, *Sarm1*^−/−^ 0.11 ± 0.02 mm^2^ vs. 0.07 ± 0.01 mm^2^, *p* < 0.0001). However, *Sarm1*^−/−^ optic nerves receiving rotenone had increased axonal density relative to wild types (Proximal optic nerve: 201,631 ± 12,619 axons/mm^2^ vs. 187,926 ± 7851 axons/mm^2^, *p* < 0.05; Distal optic nerve: 188,764 ± 13,580 axons/mm^2^ vs. 172,002 ± 7086 axons/mm^2^, *p <* 0.05). Indeed, density of axons in proximal (Figure 4c) and distal (Figure 4f) rotenone-treated *Sarm1*^−/−^ optic nerves was similar to that of nerves that had not received a rotenone insult (Proximal: rotenone-insulted *Sarm1*^−/−^ 201,631 ± 126,219 axons/mm^2^ vs. uninjected *Sarm1*^−/−^ 196,265 ± 3030 axons/mm^2^, uninjected *Sarm1*^+/+^ 200,835 ± 9874 axons/mm^2^. Distal: rotenone-insulted *Sarm1*^−/−^ 188,764 ± 13,580 axons/mm^2^ vs. uninjected *Sarm1*^−/−^ 194,208 ± 9417 axons/mm^2^, uninjected *Sarm1*^+/+^ 190,789 ± 7402 axons/mm^2^).

### 2.3. Bioenergetics of Primary Fibroblasts

**Figure 5:**
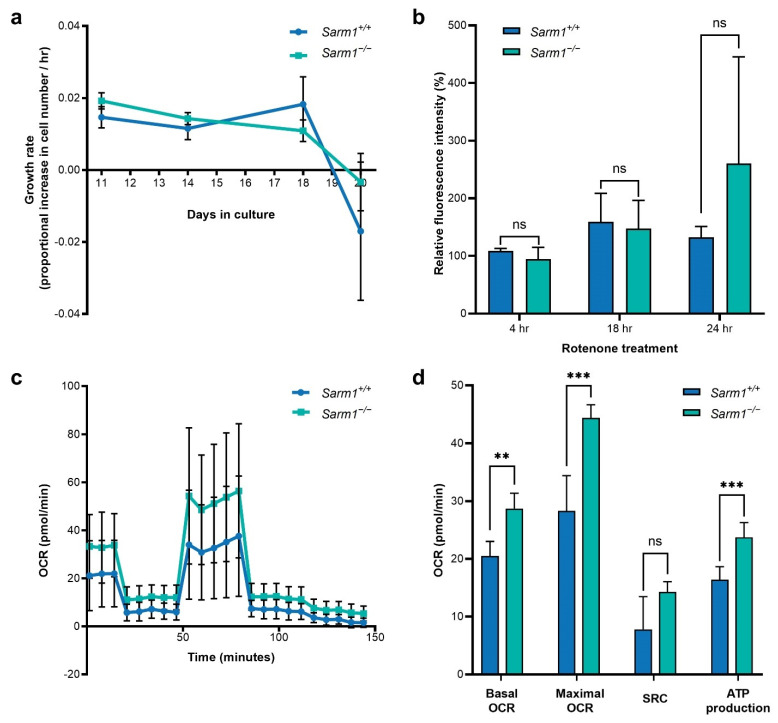
Growth and bioenergetics of *Sarm1^+/+^* and *Sarm1*^−/−^ primary fibroblasts. (**a**) Growth rate over time. Mean growth rate (proportional increase in cell population over time) for each genotype is plotted against days in culture. *Sarm1^+/+^* (*n* = 5) shown in blue, *Sarm1*^−/−^ (*n* = 5) shown in green. Growth rates were similar in both genotypes, with populations beginning to contract after 18 days in culture. (**b**) ROS accumulation following rotenone treatment. *Sarm1^+/+^* (*n* = 4) and *Sarm1*^−/−^ fibroblasts (*n* = 5) were treated with 2.5 µM rotenone for 4, 18 and 24 h before ROS accumulation was assayed through DCFDA assay. ROS accumulated to a similar degree in both genotypes (4 h: *Sarm1^+/+^* 109.11 ± 4.02% fluorescence relative to control vs. *Sarm1*^−/−^ 94.92 ± 19.94%; 18 h: *Sarm1^+/+^* 159.09 ± 49.40% vs. *Sarm1*^−/−^ 147.35 ± 48.79%; 24 h: *Sarm1^+/+^* 132.62 ± 18.67% vs. *Sarm1*^−/−^ 260.50 ± 185.11%). (**c**,**d**) Real time measurements of OCR were taken using the Seahorse XFe96 Analyser. Oligomycin (1.0 µM), FCCP (1.0 µM), rotenone (0.5 µM) and antimycin A (0.5 µM) were injected sequentially. Basal and maximal OCR, and ATP production were increased in *Sarm1*^−/−^ fibroblasts relative to wild type (44.38 ± 2.26 pmol/min vs. 28.31 ± 6.10 pmol/min, *p* < 0.001; 14.28 ± 1.78 pmol/min vs. 7.80 ± 5.67 pmol/min, *p* < 0.05; 23.73 ± 2.57 pmol/min vs. 16.38 ± 2.62 pmol/min, *p* < 0.001); while SRC demonstrated a non-significant trend towards an increase in *Sarm1*^−/−^ fibroblasts relative to wild type. Error bars represent SD values, ** *p* < 0.01, *** *p* < 0.001, ns = non-significant.

In order to investigate whether there may be intrinsic differences between wild type *Sarm1^+/+^* and *Sarm1*^−/−^ cells, primary fibroblasts were extracted from age and sex-matched mice (*n =* 5) of each genotype and cultured for 21 days. Growth rates of cells of each genotype did not differ significantly (Figure 5a).

Rotenone induces mitochondrial dysfunction through inhibition of complex I, blocking oxidative phosphorylation and leading to production of ROS. A DCFDA assay was used to examine accumulation of ROS in rotenone-treated fibroblasts and assess whether ROS accumulates to the same extent in each genotype (*n =* 4 *Sarm1^+/+^*; *n =* 5 *Sarm1*^−/−^). No difference was observed between fibroblast cells from the two genotypes from 4–24 h post-treatment with 2.5 µM rotenone (4 h: *Sarm1^+/+^* 109.11 ± 4.02% relative fluorescence intensity vs. *Sarm1*^−/−^ 94.92 ± 19.94%; 18 h: *Sarm1^+/+^* 159.09 ± 49.40% vs. *Sarm1*^−/−^ 147.35 ± 48.79%; 24 h: *Sarm1^+/+^* 132.62 ± 18.67% vs. *Sarm1*^−/−^ 260.50 ± 185.11%; Figure 5b).

We next examined mitochondrial function with a Seahorse MitoStress test (Figure 5c,d). Cells (from *n =* 5 mice for each genotype) were seeded at equal density on day 21 of culture and analysed on day 22. Of note, *Sarm1*^−/−^ cells had significantly higher basal OCR (28.71 ± 2.68 pmol/min vs. 20.51 ± 2.51 pmol/min, *p <* 0.01) maximal OCR (44.38 ± 2.26 pmol/min vs. 28.31 ± 6.10 pmol/min; *p* < 0.001), SRC (14.28 ± 1.78 pmol/min vs. 7.80 ± 5.67 pmol/min; *p* < 0.05) and ATP production (23.73 ± 2.57 pmol/min vs. 16.38 ± 2.62 pmol/min; *p* < 0.001) than wildtype *Sarm1^+/+^* cells. There was no difference in extracellular acidification rate between the genotypes. Mitochondrial copy number and relative expression of the mitochondrial gene *Cox1* was similar in both genotypes (Figure S2).

## 3. Discussion

Many currently untreatable ocular conditions feature degeneration of the optic nerve, resulting in loss of connectivity between the retina and the brain. While protection of the RGC somas in the retina is vital, protection of the RGC axons that form the optic nerve is also critical if vision is to be preserved. We aimed to ascertain whether deletion of SARM1, a prodegenerative NADase, is protective in vivo against rotenone-induced mitochondrial dysfunction in RGCs and subsequent deterioration in vision. Notably, we have obtained consistent significant benefit in spatial vision in *Sarm1*^−/−^ mice at multiple time points post rotenone insult. Importantly, *Sarm1*^−/−^ mice insulted with rotenone did not suffer significant loss of spatial vision, indicating maintenance of connectivity and function of RGCs. PhNR was not significantly reduced in *Sarm1*^−/−^ mice following rotenone insult, unlike in wild type *Sarm1^+/+^* control mice. In addition to the significant preservation of RGC function in *Sarm1*^−/−^ mice, while there was significant RGC loss following rotenone insult in wild type and *Sarm1*^−/−^ retinas, *Sarm1*^−/−^ retinas insulted with rotenone showed substantial preservation of RGCs compared to wild type controls (Figure 3b), indicating significant protection of RGCs by SARM1 ablation.

Given the inhibition of complex I and oxidative stress resulting from rotenone insult [43,44,45,46,47,48,49], it is not surprising that we observed significant RGC death in rotenone-insulted wild type and *Sarm1*^−/−^ retinas. Rotenone insult resulted in a non-significant decrease in cell number in the inner and outer nuclear layers (Figure S1). While RGC death occurred in both genotypes (Figure 3b), we observed higher numbers of RGC cell bodies in *Sarm1*^−/−^ retinas following insult than in controls, indicating protection by SARM1 ablation against progressive degeneration of RGCs. It is perhaps notable that protection of RGC function despite RGC loss has been reported previously [58]. Optic neuropathies tend to be characterised by injury to the axons of RGCs, which subsequently start to degenerate, resulting in a loss of connectivity between the retina and brain, with ensuing somal degeneration [7,13]. While most optic neuropathies cannot be reversed [7], some benefit may be obtained from intervention delaying damage to the optic nerve from progressing to the cell body [59]. Critically, we have shown significant protection of axonal density in the optic nerve of *Sarm1*^−/−^ mice receiving rotenone (Figure 4c,f). Moreover, we have demonstrated in vivo significant rescue of functional connectivity between the retina and the brain in *Sarm1*^−/−^ mice treated intravitreally with a rotenone insult, as demonstrated through preservation of spatial vision in both sexes over time compared to controls (Figure 1) and maintenance of PhNR (Figure 2).

*Sarm1* deletion has been found previously to be protective against RGC death and axon degeneration in a neuroinflammatory model of glaucoma and in a mouse model of amyotrophic lateral sclerosis (ALS) [60,61]. In the optic nerve crush model, *Sarm1* ablation protected against RGC axon degeneration but not soma death [36,62]. In contrast, deletion of the pro-apoptotic gene *Bax* does not protect against RGC loss in an NMDA-induced excitotoxicity model of glaucoma but does protect against death after optic nerve crush, suggesting there may be distinct degeneration pathways at play in different disease settings and that a combinational approach protecting axons and soma may be desirable [5,14,15]. In the current study, we have demonstrated both significant preservation of RGC function and protection of both RGC axons and cell bodies in *Sarm1*^−/−^ retinas.

As stated above, RGC death occurred in both genotypes following rotenone insult, however there was increased RGC density in *Sarm1*^−/−^ retinas, with remaining RGCs in *Sarm1*^−/−^ retinas more evenly distributed than in wild type (Figure 3). In contrast, there was increased variation in distances between neighbouring RGCs in wild type retinas insulted with rotenone, indicating that some areas within the one retina display sparse RGCs while others had a higher density (Figure 3c). This may contribute to the preservation of spatial vision with spatial frequency readings remaining high in rotenone-insulted *Sarm1*^−/−^ retinas despite significant RGC death. Optokinetic response measurements enable quantification of the spatial vision of the mouse. Higher spatial frequency readings indicate that the mouse is able to differentiate between increasingly narrower lines in a sine wave grating [63,64]. As rotenone-insulted *Sarm1*^−/−^ retinas retained RGCs across the retina representing much of the visual field, it is suggestive that there is still signal arising from most parts of the retina to the brain, allowing OKR measurements and associated spatial vision to remain high (Figure 1 and Figure 3c). In contrast, rotenone-insulted wild type retinas displayed large areas devoid of RGCs (Figure 3c).

Notably, we have consistently demonstrated preservation of visual function in *Sarm1*^−/−^ mice treated with rotenone in both sexes over a prolonged period of time. Visual function remains high in *Sarm1*^−/−^ mice after 4 months, demonstrating that the beneficial effects of *Sarm1* deletion is maintained over time and that damage to axons is not merely delayed relative to wild type. Several forms of optic neuropathy feature vision loss that is reversible if treated early, before the injured axon begins to degenerate [7,8]. The long-lasting functional benefit afforded by *Sarm1* deletion highlights the importance of preservation of RGC axons. Furthermore, both sexes benefit equally from absence of *Sarm1*, which is particularly relevant in the context of emerging differences between sexes for many disorders and treatment regimes [53,54,55,56,65]. While a significant number of RGCs succumbed to rotenone-induced injury, increased RGCs in *Sarm1*^−/−^ retinas coupled with a trend towards a more even distribution of RGCs across the retina results in preservation of signal from more of the retina. Moreover, significant protection of axonal density in the optic nerve maintains the connection between the retina and the visual cortex.

The retina has enormous energy demands and so is highly dependent on functional mitochondria. The neuronal architecture of RGCs leaves them particularly vulnerable, with their axons running unmyelinated along the retinal nerve fibre layer before leaving the retina to form the optic nerve where they are myelinated [1,66,67]. Furthermore, these axons extend from the retina to the visual cortex and must meet energy requirements over this distance. As such, perturbations to mitochondrial function may be more likely to affect these cells than others. We used rotenone to inactivate complex I and induce mitochondrial dysfunction to establish if SARM1 absence may be protective in vivo in the mouse retina. Rotenone has been shown to activate SARM1, including at sub-critical levels [50]. As RGCs represent less than 1% of retinal cells and are challenging to purify, requiring large numbers of animals, we extracted primary fibroblasts from wild type and *Sarm1*^−/−^ mice to evaluate mitochondrial function. Indeed, primary fibroblasts from patients with neurodegenerations are commonly used to assess cellular bioenergetics [68,69].

We found no major differences in growth rate between wild type and *Sarm1*^−/−^ cells. Additionally, we found no difference in the accumulation of ROS between the two genotypes when insulted with rotenone (Figure 5c). This observation is corroborated by another study that reported similar levels of ROS accumulation in wild type and *Sarm1*^−/−^ brain neurons [14]. Despite insult with oxidative stressors, *Sarm1*^−/−^ brain neurons were protected from degeneration suggesting that SARM1 may function downstream from ROS accumulation and that *Sarm1* ablation can be protective despite severe mitochondrial dysfunction and ROS accumulation [14].

Analyses of mitochondrial health in fibroblasts using the Seahorse MitoStress test revealed significant differences between the two genotypes with significantly increased basal and maximal OCRs and ATP generation in *Sarm1*^−/−^ fibroblasts, and a non-significant increase in SRC. To our knowledge, not all of these parameters (Figure 5e) have been evaluated between these two genotypes previously. However, increased OCR has been reported in *Sarm1*^−/−^ brain neurons relative to wild type following axotomy, while *Sarm1* overexpression has been found to decrease basal and spare respiratory capacity [40]. ATP production has been found to be increased in *Sarm1*^−/−^ neurons in vitro and decreased with *Sarm1* overexpression [14,36,40]. In the current study, multiple parameters were evaluated including the basal and maximal OCR, the SRC and ATP generation, with all parameters pointing towards increased bioenergetic profiles in *Sarm1*^−/−^ primary fibroblasts. As the relative mitochondrial copy number of *Sarm1*^−/−^ cells was similar to wild type, increased bioenergetics suggests more efficient mitochondria rather than a higher number of mitochondria in these cells (Figure S2).

Given that we have observed significant functional and histological benefit in the rotenone-induced retinal degeneration mouse model, and in addition, have found a significantly increased bioenergetic profile in primary *Sarm1*^−/−^ fibroblasts compared to wild type *Sarm1^+/+^* cells, it is highly suggestive that the increased bioenergetic profile contributes to the protective effect observed in vivo in retina. This bioenergetic surplus in *Sarm1*^−/−^ cells, in conjunction with the absence of SARM1 NADase activity, which has been previously well described in *Sarm1*^−/−^ mice and shown to be protective in *Sarm1*^−/−^ neurons [24,29,37,70], may underlie the preservation of retinal function observed in the current study. Regardless of the mechanism, it is clear that *Sarm1* ablation can protect in vivo in this mouse model for complex I dysfunction, a hallmark of many neurodegenerative conditions including a variety of ocular disorders [71,72,73,74,75].

## 4. Materials and Methods

### 4.1. Animals and Intravitreal Injections

All animal work was performed in accordance with the European Union (Protection of Animals used for Scientific Purposes) Regulations 2012 (S.I. no. 543 of 2012) and the Association for Research in Vision and Ophthalmology (ARVO) statement for the use of animals. Wild type C57BL/6J (JAX stock 000664) and *Sarm1*^−/−^ (B6.129X1-Sarm1tm1Aidi/J; JAX stock no. 018069) mice were housed in a specific pathogen free (SPF) facility under a 12-h light/dark cycle, with access to water and food ad lib. Pupils were dilated with 1% tropicamide and 2.5% phenylephrine. Mice were anaesthetised by intraperitoneal injection of ketamine and medetomidine (45.45 mg/kg body weight; 0.45 mg/kg body weight, respectively). Under topical anaesthesia (Amethocaine), a puncture was made in the sclera and a 26-gauge microneedle, attached to a 10 µL Hamilton syringe, inserted through the puncture. 0.6 μL 2 mM (1.2 nmol) or 2.5 mM rotenone (1.5 nmol) in dimethyl sulfoxide (DMSO, vehicle) was slowly injected into the vitreous. Following injection into each eye, anaesthetic reversing agent (2.27 mg/kg body weight, atipamezole) was delivered by intraperitoneal injection. The body temperature of mice was maintained during recovery.

### 4.2. Optokinetic Response Measurements

Mice underwent optokinetic analysis 2 and 4 months post-rotenone, as previously described [44,46]. Optokinetic response (OKR) spatial frequency thresholds were measured using a virtual optokinetic system (VOS, OptoMotry, CerebralMechanics, Lethbridge, AB, Canada; [64]). Four computer monitors facing inwards created a virtual reality chamber, with the unrestrained mouse on a platform in the centre. A video camera, pointing down at the animal, provided real-time video feedback. OptoMotry measures spatial frequency thresholds by projecting a virtual cylinder covered with a moving sinewave grating onto the monitors. Changing direction of the grating enables the threshold of each eye to be determined [53]. As the mouse was not restrained, the experimenter centred the virtual cylinder on the mouse’s head and observed whether the mouse tracked the grating with its head and neck. The spatial frequency of the grating at 100% was gradually increased until the point at which the mouse no longer tracked the grating—its spatial frequency threshold. OKRs for each mouse were measured 3–4 times on separate days at each time point. Combined readings (i.e., the average reading of the left and right eyes) for each animal were averaged and SD values calculated.

### 4.3. Photopic Negative Response

Six weeks following rotenone treatment, photopic negative response (PhNR) was measured. Mice were anaesthetised as above. The PhNR, the negative deflection following the b-wave response, was evaluated under photopic conditions using a Roland Consult RetiScan ERG RetiPort electrophysiology unit ((Roland Consulting, Brandenburg-Wiesbaden, Germany). Detection was enhanced with an orange filter. PhNRs of contralateral eyes were recorded simultaneously from both eyes by means of goldwire electrodes (Roland Consulting, Brandenburg-Wiesbaden, Germany). Cone-isolated responses were recorded to the maximal intensity flash (−25 dB maximal intensity where maximal flash intensity was 3 candelas/m^2^/s). Resulting waveforms were marked according to International Society for Clinical Electrophysiology of Vision conventions.

### 4.4. Histology

Eyes were enucleated, fixed overnight in 4% paraformaldehyde in PBS before the retinas were removed from the eyecup, and processed for immunohistochemistry. Immunohistochemistry was performed as previously described [76]. Retinas were incubated with anti-BRN3A primary antibody (Synaptic Systems 411003, Goettingen, Germany) for 3 days at 4 °C. Samples were washed in PBS and stained with secondary antibodies conjugated with Alexa Fluor 488, Cy3 (Jackson ImmunoResearch Laboratories, PA, USA; 1/400 dilution) for 3 days at 4 °C. Retinas were whole-mounted using Hydromount (National Diagnostics, UK). Images were taken using an Olympus IX83 inverted motorised microscope (Mason Technology, Ireland) featuring a SpectraX LED (Lumencor, Mason Technology, Ireland) and an Orca-Flash4.0 LT PLUS/sCMOS camera (Hamamatsu, Mason Technology, Ireland), as previously described [77]. Lateral frames were stitched together in Olympus CellSens software (Version 1.9, Waltham MA, USA). RGCs were counted using 2D-deconvolution, manual threshold and object size filters, with the same settings applied to all images.

Scatter of RGCs across the retina was analysed by calculating the distances between neighbouring RGCs. Even distribution of RGCs across the retina was expected to result in homogenous measurements, while clusters and sparse zones in the same retina would result in a variety of distances. 4–8 samples of set size were taken for each retina, totalling 70–80% of the area of the retina. Samples were imported into ImageJ (1.5c, National Institute of Health, MD, USA) and processed using the 2D Particle Distribution tool of the BioVoxxel toolbox, with the same settings applied [57].

Optic nerves from fixed eyes were washed in PBS and cryoprotected in 10%, 20% and 30% sucrose. The 2 mm segments of proximal and distal optic nerve, from the eye and brain side of the nerve respectively, were embedded in OCT and frozen. Samples were sectioned (7 μm) on a Leica CM1950 and thaw-mounted on Polysine slides (ThermoFisher, Waltham MA, USA). Slides were incubated in anti-NF200 antibody (Sigma N4142; 1/300 dilution) overnight at 4 °C before incubation with secondary antibodies conjugated with Alexa Fluor 488, Cy3 (Jackson ImmunoResearch Laboratories, West Grove, PA, USA; 1/400 dilution) for two hours at room temperature. Nuclei were counterstained with DAPI. Coverslips were mounted using Hydromount. Images were taken as above and axons were counted using 2D deconvolution, particle separation, manual threshold and object size filters, with the same settings applied to all images. Quantification was performed on 3–5 sections per sample.

### 4.5. Isolation of Primary Fibroblasts from Mouse Tails

Protocol for isolation of fibroblasts was adapted from Khan and Gasser (2016) [78]. Tails from 1 month old C57BL/6J (n = 5) and *Sarm1*^−/−^ (n = 5) mice were placed in 70% ethanol for 3 h. Tails were then dried in a laminar flow cabinet, cut into small (~3 mm) sections and digested with 1.2 mg/mL pronase (Sigma Aldrich, Dublin, Ireland) and 2.5 mg/mL collagenase (Sigma Aldrich) for 90 min at 37 °C in an orbital shaker set at 200 rpm. Digested tissue was strained through a 70 µm cell strainer (ClearLine, Scientific Laboratory Supplies, Dublin, Ireland) into 5 mL media (RPMI, 5% L-Glut, 20% FBS, 1% Pen/Strep, 50 µM 2-mercaptoethanol, 100 µM asparagine). Cells were transferred to a 15 mL tube and centrifuged at 580 g for 7 min. Supernatant was removed and cell pellets resuspended in 5 mL media before a second centrifugation. Cell pellets were resuspended in 10 mL media and plated onto 10 cm dishes with 10 µL amphotericin B (Fisher Scientific, Dublin, Ireland). Media was changed 3 days post-extraction. Cells were first passaged 7 days post-extraction and maintained at 1:3 splits until passage 5–6.

### 4.6. Growth Assays

At each passage, cells were counted using a haemocytometer. Growth rate (*gr*) was calculated as $gr=\frac{ln\left(\frac{N_{t}}{N_{0}}\right)}{t}$, where $N_{t}$ and $N_{0}$ are cell counts at harvest and seeding respectively, and plotted against time to assess changes in rate of growth over time.

### 4.7. Analysis of ROS Accumulation

The 6 × 10^3^ primary fibroblasts extracted from *n* = 5 *Sarm1*^−/−^ and *n* = 5 wild type mice were seeded into a black-walled 96 well plate. The following day, cells were treated with rotenone (final concentration 2.5 µM) for 4, 18 or 24 h, with four replicates for each time point for each cell line. ROS accumulation was examined by 2′,7′ –dichlorofluorescein diacetate (DCFDA) assay. Briefly, cells were incubated in CellROX Green Reagent (final concentration 5 µM; Life Technologies, CA USA) in glucose free media without phenol red for 30 min at 37 °C before washing with PBS. Signal was read using a FluorOptima plate reader.

### 4.8. Analysis of Mitochondrial Function

The 7.5 × 10^3^ primary fibroblasts isolated from mouse tail were seeded into an Agilent XFe96 Seahorse plate (*n =* 5 *Sarm1*^−/−^ and *n* = 5 *Sarm1^+/+^* wild type mice). Six replicates were seeded of each. A mitochondrial stress test was performed the following day according to the manufacturer’s protocols to compare basal OCRs and maximal SCRs. Final well concentrations of oligomycin, FCCP, rotenone and antimycin A were 1.0 µM, 1.0 µM, 0.5 µM and 0.5 µM respectively. Tail fibroblasts were assayed on day 22 post-isolation.

### 4.9. Statistical Analyses

All statistical analyses were performed using GraphPad Prism (version 9.3, GraphPad Software, La Jolla, CA, USA, www.graphpad.com). For experiments with two groups, two-tailed unpaired *t*-test with Welch’s correction was used. For experiments involving two factors (e.g., genotype and time, genotype and treatment), two-way ANOVA was used with Tukey’s multiple comparison post-hoc test. A *p*-value < 0.05 was considered significant.

## Data Availability

The datasets used and/or analysed during the current study are available from the corresponding author on reasonable request.

## Supplementary Materials

The following are available online at https://www.mdpi.com/article/10.3390/ijms23031606/s1.
