# Supplementary material for: SARM1 Ablation Is Protective and Preserves Spatial Vision in an In Vivo Mouse Model of Retinal Ganglion Cell Degeneration
Source: Int J Mol Sci. 2022 Jan 30;23(3):1606. doi: 10.3390/ijms23031606 (PMC8835928; doi:10.3390/ijms23031606)
Supplement: Supplementary file 1 [file ijms-23-01606-s001.zip › ijms-1459508-supplementary.pdf]

International Journal of
Molecular Sciences

MDPI

1. Materials and methods

1.1. Quantification of Nuclei in Inner and Outer Nuclear Layers in Rotenone-Insulted Retinal Sections

Eyes (n = 3) were enucleated, fixed overnight in 4% paraformaldehyde in PBS before dissection and cryopreservation before cryosectioning at 12 µm. Sections were stained with DAPI and imaged. FeatureJ edge separation filters were applied in ImageJ. Cells were manually marked in Adobe Photoshop and marked cells counted in ImageJ.

1.2. Analysis of Mitochondrial Copy Number

The 1x10^5 primary fibroblasts were pelleted and frozen (n = 5 *Sarm1*$^{-/-}$ and n = 5 *Sarm1*$^{+/+}$). DNA was isolated using Qiagen Blood and Tissue Kit. Real time quantitative PCR was used to determine the quantity of mitochondrial DNA (mtDNA) relative to nuclear DNA (nDNA). A fragment of the mitochondrial *16S rRNA* gene was amplified using primers 5′-CTAGAAACCCCGAAACCAAA-3′ (forward) and 5′-CCAGCTATCACCAAGCTCGT-3′ (reverse). Primers targeting the nuclear *Cfh* gene were 5′-GCACATAAATAGCTGGATGGACCTG-3′ (forward) and 5′-TTGTTCTGACCACGAGCC-3′ (reverse). 12.5 ng DNA, 0.5 µM of each primer and 2x SYBR Master Mix comprised each 20 µL reaction. Reactions were carried out in triplicate in a StepOne™ Real-Time PCR System (Applied Biosciences). A 6-point standard curve of serial dilutions (1:5) was used to determine relative amounts of mtDNA and nDNA. Mitochondrial copy number was determined by dividing the relative amount of mtDNA by the relative amount of nDNA.

1.3. Expression of Mitochondrial Gene Cox1

The 1x10^5 primary fibroblasts were pelleted and frozen (n = 5 *Sarm1*$^{-/-}$ and n = 4 *Sarm1*$^{+/+}$). RNA was isolated using Qiagen RNeasy Mini Kit. Relative expression levels of *Cox1* were determined through RT-qPCR. *Cox1* primers were 5′-TCGGAGCCCCAGATATAGCA-3′ (forward) and 5′-TTTCCGGCTAGAGGTGGGTA-3′ (reverse). Primers targeting transcripts from the housekeeping gene *β-actin* were 5′-AGAGCAAGAGAGGCATCC-3′ (forward) and 5′-TCATTGTAGAAGGTGTGGTGC-3′ (reverse). Amounts of 8 ng RNA, 0.5 µM of each primer, 0.15 µL RT Enzyme and 2x SYBR Master Mix comprised each 20 uL reaction. Reactions were carried out in triplicate in a StepOne™ Real-Time PCR System (Applied Biosciences). A 5-point standard curve of serial dilutions (1:5) was used to determine relative amounts of each transcript. Relative expression was determined by dividing the relative amount of *Cox1* by the relative amount of *β-actin*.

2. Results

2.1. Effect of Rotenone Insult on Inner and Outer Nuclear Layers

To assess the impact of rotenone insult on inner and outer nuclear layers (INL and ONL), wild type eyes (n = 3) insulted with rotenone and controls were cryosectioned and stained with DAPI. Nuclei in the INL and ONL were counted. While the number of nuclei in both layers were decreased, this was not significant (INL: 100 ± 18% vs. 69 ± 14%; ONL: 100 ± 5% vs. 93 ± 11%; Figure S1). There was a non-significant trend towards decreased cell number in the INL (p = 0.08).

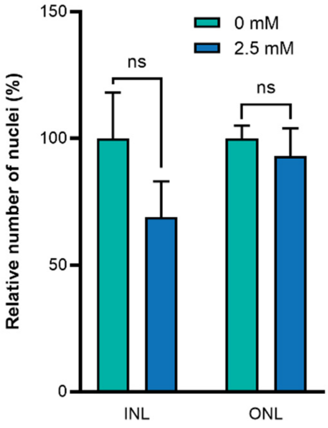

Figure S1. Number of nuclei in inner and outer nuclear layers following rotenone insult. Bar charts represent the mean relative number of nuclei counted manually in the inner and outer nuclear layers (INL and ONL respectively). Rotenone insult resulted in a non-significant decrease in number of nuclei in both layers (INL: 100±18% vs. 69 ± 14%; ONL: 100 ± 5% vs. 93 ± 11%), with a trend towards significance in the INL (p = 0.08). Error bars represent SD.

2.2. Number of Mitochondria and Expression of Mitochondrial Components is Similar in Sarm1$^{+/+}$ and Sarm1$^{-/-}$ Primary Fibroblasts

To assess whether the increased mitochondrial function observed was due to *Sarm1*$^{-/-}$ fibroblasts possessing more efficient or more plentiful mitochondria, relative mitochrondial copy number was determined through real-time quantitative PCR. There was no difference in the relative mtDNA/nDNA ratio between *Sarm1*$^{+/+}$ and *Sarm1*$^{-/-}$ genotypes (1.20 ± 0.32 vs. 1.08 ± 0.19; Figure S2a).

Relative expression of mitochondrial gene *Cox1*, whose protein product is a subunit of complex IV of the electron transport chain, was determined through RT-qPCR. Relative expression of *Cox1* was similar in *Sarm1*$^{+/+}$ and *Sarm1*$^{-/-}$ primary fibroblasts (1.30 ± 0.28 vs. 1.39 ± 0.72; Figure S2b).

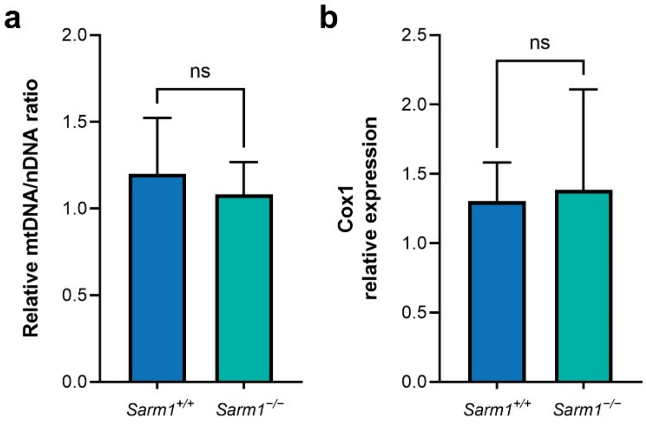

Figure S2. Mitochondrial copy number and expression of mitochondrial component *Cox1*. (**a**) Relative mitochondrial copy number. Bar charts represent mean relative mtDNA/nDNA ratio. Relative mtDNA/nDNA ratio was similar in wild type and *Sarm1*$^{-/-}$ primary fibroblasts (1.20 ± 0.32 vs. 1.08 ± 0.19). (**b**) Relative expression of mitochondrial gene *Cox1* was similar in both genotypes (1.30 ± 0.28 vs. 1.39 ± 0.72).
